# Pericardial effusion in patients with chronic kidney disease: A two-center study

**DOI:** 10.1371/journal.pone.0302200

**Published:** 2024-06-06

**Authors:** Vahid Eslami, SeyedehFatemeh Mousavi, Rana Irilouzadian, Hediyeh Baghsheikhi, Mehrdad Jafari Fesharaki, Shiva Samavat

**Affiliations:** 1 Cardiovascular Research Center, Shahid Modarres Hospital, Shahid Beheshti University of Medical Sciences, Tehran, Iran; 2 Clinical Research Development Center, Shahid Modarres Educational Hospital, Shahid Beheshti University of Medical Sciences, Tehran, Iran; 3 School of Medicine, Shahid Beheshti University of Medical Sciences, Tehran, Iran; 4 Burn Research Center, Iran University of Medical Sciences, Tehran, Iran; 5 USERN Office, Shahid Beheshti University of Medical Sciences, Tehran, Iran; 6 Department of Cardiology, School of Medicine, Shahid Beheshti University of Medical Sciences, Tehran, Iran; 7 Chronic Kidney Disease Research Center (CKDRC), Shahid Labbafinejad Medical Center, Shahid Beheshti University of Medical Sciences (SBMU), Tehran, Iran; Phramongkutklao College of Medicine, THAILAND

## Abstract

**Background and aims:**

Pericardial effusion (PE) is a prevalent form of pericardial involvement in chronic kidney disease (CKD). This study aims to investigate the clinical and laboratory features associated with PE severity in patients with CKD.

**Methods:**

In this cross-sectional study, we examined the medical records of patients admitted to tertiary hospitals with International Classification of Diseases 10th Revision (ICD-10) codes associated with CKD and PE. We included 112 CKD patients in stage 4 and 5 non-dialysis (ND) with PE for assessing the clinical and laboratory features of severity.

**Results:**

Patients were divided into two categories based on the severity of PE. Seventy-two patients had mild and 40 had moderate and severe PE. Univariate analysis of demographic and laboratory features on the date of admission demonstrated that chest pain, dyspnea, serum albumin, and neutrophil-to-lymphocyte ratio (NLR) are associated with the severity of PE. The univariate analysis on the date of echocardiography showed significantly higher white blood cell count (WBC), neutrophil count (percentage and absolute count), and NLR, along with significantly lower lymphocyte percentage and serum albumin among patients with moderate and severe PE. In the multivariable analysis of laboratory features, on admission hypoalbuminemia (p-value = 0.014, OR = 4.03, CI: 1.32–12.25) and NLR greater than 5.5 (p-value = 0.015, OR = 4.22, CI: 1.32–13.50) were significantly associated with moderate and severe PE. In a parallel matter, at the time of echocardiography hypoalbuminemia (p-value = 0.004, OR = 5.38, CI: 1.74–16.65) and neutrophilia (p-value = 0.005, OR = 7.94, CI: 1.89–33.44) were significantly associated with moderate and severe PE.

**Conclusion:**

Despite advancements in the diagnosis and treatment of CKD, PE is still a concerning issue in these patients. This study revealed that hypoalbuminemia, neutrophilia, and NLR greater than 5.5 could be predictive factors of moderate and severe PE in CKD patients with PE. Further prospective study with larger sample size is needed to confirm these results.

## Introduction

Chronic kidney disease (CKD) is among the challenging health-related issues with growing concern. In 2019, more than 1.4 million deaths were attributable to CKD globally [1]. Pericardial syndrome, including pericarditis, pericardial effusion (PE), cardiac tamponade and the less common chronic constrictive pericarditis, can further complicate CKD and end-stage renal disease (ESRD) cases [2]. Uremic pericarditis occurs before or within the first eight weeks of the dialysis initiation and without timely management, is associated with poor outcomes in patients with CKD [3]. Dialysis-associated pericarditis is a term applied when it manifests more than 8 weeks following the commencement of dialysis. Nevertheless, some evidence suggests that there may not be a clear-cut demarcation between these conditions [4].

The typical pericardial sac holds between 10 to 50 ml of pericardial fluid, functioning as a plasma ultrafiltrate that provides lubrication between the layers of the pericardium. If there is an accumulation of transudative or exudative fluid exceeding 50 mL, it is considered abnormal and referred to as PE [2, 5].

Acute pericarditis and PE might occur simultaneously or alone. Acute pericarditis is characterized by inflammatory condition and is diagnosed based on meeting two or more criteria, including chest pain, pericardial friction rub, electrocardiographic changes, and the presence of PE. However, it tends to be less symptomatic in ESRD patients [2, 6]. Conversely, PE is not always associated with inflammation and, in a significant number of ESRD patients, could be secondary to volume overload [2]. Notably, the majority of cases of PE remain asymptomatic until they reach severe conditions or tamponade develops [7].

The reported prevalence of pericardial disease in patients with CKD varies significantly in literature, possibly due to disparities in reporting time frames, diagnostic and treatment methods, facilities and specialties available at different hospitals. In addition, some studies have lacked clarity in defining uremic and dialysis-associated pericarditis and PE [4]. The prevalence of PE among individuals with CKD, without specifying a time frame relative to dialysis, have been reported to range from 1.9 to 62% [4, 7–14]. Among patients who have undergone pericardial fluid drainage, the etiology of PE was attributed to uremia in 3.78% to 67% of cases; notably, a majority of these studies encompassed patients under maintenance dialysis [15–28].

Due to the paucity of data on diagnosis and severity predictors of pericardial disease among the CKD population in recent years [3], this study aims to address clinical and laboratory features associated with the severity of PE in patients with CKD in two referral hospitals in Tehran, Iran.

## Methods

### Study design and setting

In this retrospective cross-sectional study, we recruited stage 4 and 5 non-dialysis (ND) CKD patients with PE who were admitted to Modarres and Labbafinejad hospitals from March 2011 through December 2021.

### Participants

**Fig 1:**
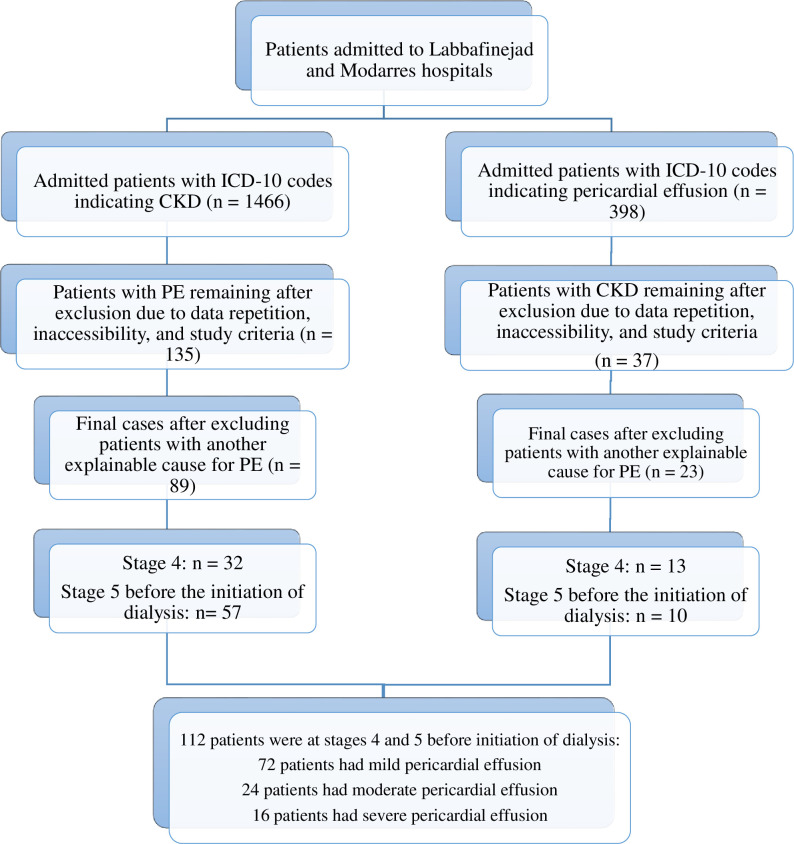
Patient selection flowchart based on the ICD-10 codes of admitted patients.

The study involved a thorough review of clinical records and laboratory data for 1,466 adult patients admitted with International Classification of Diseases 10th Revision (ICD-10) codes indicating CKD, along with 398 patients bearing ICD-10 codes related to PE, irrespective of its underlying cause. Specifically, patients in CKD stages 4 and 5ND with coexisting PE were recruited for the study. The exclusion criteria were as follows: concomitant infectious disease, systemic autoimmune and inflammatory diseases, neoplasms and paraneoplastic syndromes, myocardial infarction within a month, cardiac surgery within a month, aortic dissection and myocarditis, uncontrolled hypothyroidism, drugs such as minoxidil and hydralazine that can result in pericardial involvement. Patient selection is shown in Fig 1, and the reasons of being excluded are demonstrated in S1 and S2 Tables.

### Variables and data collection

Demographic and anthropometric data, clinical characteristics, and laboratory findings were gathered with a pre-designed data collection form. Two independent researchers separately reviewed and double-checked the obtained data. Clinical symptoms were reported at the time of admission. The stage of kidney disease is calculated using creatinine at the time of admission with CKD-EPI 2021 equations for glomerular filtration rate (GFR) [1]. Systematic documentation of laboratory findings was performed both upon admission and during the initial echocardiography assessment. This sequencing was chosen to ensure the proximity of laboratory data on date of echocardiography to the actual measurement of PE. However, it is acknowledged that interventions for symptomatic therapy may lead to alterations in some of these parameters. As a result, we also collected data regarding the date of admission.

In M-mode echocardiography, a persistent echo-free zone between the epicardium and parietal pericardium is evident throughout the cardiac cycle. When this separation is exclusively observed during systole, it indicates a normal or clinically inconsequential amount of pericardial fluid, often referred to as trivial PE. Conversely, the presence of this distinction in both systole and diastole is suggestive of effusions exceeding 50 ml (small PE) [5]. In accordance with the criteria set by the American Society of Echocardiography (ASE) for transthoracic echocardiography (TTE), PE is typically classified into three categories: mild (PE size less than 10 millimeters), moderate (PE size 10–20 millimeters), and severe (PE size more than 20 millimeters) [2, 7]. In our study, we adapted the standard ASE classification to better align with our aim of identifying clinically significant PE and considering the distribution of our patient population. Consequently, we categorized participants into two groups: a mild PE group and a combined moderate/severe PE group. We categorized variables for clearer interpretation, hypoalbuminemia was defined as serum albumin less than 3.5 g/dl and neutrophilia as the absolute neutrophil count above 7700 cells [29]. Additionally, we determined that the optimum cut-off point for the neutrophil-to-lymphocyte ratio (NLR) from our dataset was 5.5.

### Statistical analysis

In our study, we used frequency and percentage to describe qualitative variables, and for quantitative variables, we employed the median along with the interquartile range (IQR). The normal distribution of data was assessed using the Shapiro-Wilk test. Quantitative variables with a normal distribution were analyzed using the independent t-test, while those with a non-normal distribution were assessed using the Mann-Whitney U test to compare subgroups of PE. The chi-square and Fisher’s exact tests were used to evaluate the association between qualitative variables and PE.

Univariate and multivariable binary logistic regression was conducted to calculate the crude and adjusted odds ratios of factors related to the severity of PE.

To mitigate the risk of overfitting, especially given the sample sizes in the moderate and severe effusion groups, we selected significant variables from univariate analyses for inclusion in multiple regression. The multivariable analysis was confined to laboratory parameters deemed clinically significant or demonstrating lower p-values, due to observed clinical and statistical collinearity among some variables. Before undertaking multiple regression, the chi-square test was applied to evaluate collinearity among independent qualitative variables, with all such variables showing p-values greater than 0.05, indicating no collinearity. The variance inflation factor (VIF) was utilized to assess collinearity within the final regression model, with no variables exceeding a VIF of 2, ensuring no significant multicollinearity was present. The P-value of 0.05 was considered as the level of statistical significance. All analyses were done using the Statistical Package for Social Science (SPSS) version 26 and R version 4.1.2.

### Ethical consideration

The study was designed and performed according to the principles of the Declaration of Helsinki. The research ethics committee of Shahid Beheshti University of Medical Sciences approved the study protocol (ethical code: IR.SBMU.1401.607).

## Results

In this study, out of 1864 patients admitted under ICD-10 codes relevant to CKD/PE, 112 individuals with CKD stages 4 and 5ND, who exhibited PE closely linked to their renal condition, were carefully chosen for inclusion. The average age of these patients was 60.8±16.1 years, with an age range of 25 to 93 years, and 42% were male. Notably, 64.2% of the patients had diabetes. Based on GFR, 41.1% and 58.5% of patients were at stage 4 and 5ND, respectively. Echocardiographic findings indicated that among the patients, 72 (64.3%) presented with mild PE, while 40 (35.7%) exhibited moderate to severe PE. The comprehensive clinical and laboratory characteristics of the study participants are detailed in Table 1.

**Table 1:** The demographics, clinical characteristics, and paraclinical features of patients among all patients and comparison between groups with mild and moderate-severe pericardial effusion.

Variable**	Total (n = 112)	Mild (n = 72)	Moderate and Severe (n = 40)	P-value
Age	61.0 (53.0–73.0)	60.0 (54.2–72.7)	61.0 (51.0–77.0)	0.625
Male sex	47(42%)	31(43%)	16(40%)	0.754
Peripheral edema	70(70.7%)	50(70.4%)	20(71.4%)	0.921
Chest pain	12(12.0%)	4(5.6%)	8(28.5%)	**0.003** *
Epigastric pain	7(7.3%)	4(5.6%)	3(12.5%)	0.361
Chest or epigastric pain	19(19%)	8(11.1%)	11 (39.2%)	**0.001** *
Dyspnea	58(54.2%)	33(45.8%)	25(71.4%)	**0.013** *
Nausea & vomiting	34(35.1%)	24(33.3%)	10(40.0%)	0.547
DM	70(64.2%)	45(65.1%)	25(62.5%)	0.775
HTN	88(80.7%)	53(78.2%)	33(85.0%)	0.390
IHD/HF	42(39.3%)	30(43.5%)	12(31.6%)	0.228
KT	11(10.3%)	6(8.8%)	5(12.8%)	0.525
Hypothyroidism***	14(13.1%)	10(14.7%)	4(10.3%)	0.511
CVA	7(6.5%)	4(5.9%)	3(7.7%)	0.704
AF	4(3.7%)	2(2.9%)	2(5.1%)	0.621
Aspirin	40(44.4%)	27(42.9%)	13(48.1%)	0.643
Prednisone	8(9.1%)	4(6.5%)	4(15.4%)	0.228
HR	80.0 (75.0–90.0)	80.0 (75.0–90.7)	80.0 (76.2–89.5)	0.991
SBP (mmHg)	150.0 (130.0–179.0)	153.5 (130.0–180.0)	137.5 (120.0–176.2)	0.173
DBP (mmHg)	88.5 (80.0–100.0)	90.0 (80.0–100.0)	80.0(78.7–90.0)	0.159
GFR (ml/min)	12.0 (8.0–17.0)	12.0 (8.0–17.0)	12.5 (7.7–18.5)	0.762
CKD				1.000
Stage 4	46(41.1%)	29(40.3%)	17(42.5%)	
Stage 5	66(58.9%)	43(59.7%)	23(57.5%)	
Cr (mg/dl)	4.5 (3.2–6.5)	4.5 (3.4–6.1)	4.1 (3.1–6.7)	0.681
BUN (mg/dl)	61.2 (47.1–81.3)	62.3 (48.4–84.8)	59.8 (40.1–73.3)	0.199
Ca (mg/dl)	8.5 (7.9–9.0)	8.5 (8.1–9.0)	8.4 (7.6–8.9)	0.277
Corrected Ca (mg/dl)	8.9 (8.4–9.4)	8.9 (8.5–9.3)	9.0 (8.2–9.5)	0.858
Mg (mg/dl)	2.2 (1.9–2.5)	2.2 (2.0–2.5)	2.2 (1.9–2.5)	0.919
K (mEq/L)	4.8 (4.4–5.4)	4.8 (4.4–5.6)	4.6 (4.3–5.1)	0.202
Na (mEq/L)	139.0 (135.7–141.0)	139.0 (135.2–141.0)	139.0 (135.7–141.2)	0.784
P (mg/dl)	5.1 (4.4–6.2)	5.1 (4.5–5.9)	5.1 (4.0–6.5)	0.957
25OH VitD_3_ (ng/ml)	14.5 (9.0–22.0)	18.0 (9.0–28.0)	12.0 (8.0–18.0)	0.176
Hb (gr/dl)	9.3 (8.2–10.7)	9.4 (8.4–10.5)	9.1 (8.1–10.9)	0.930
Hct (%)	29.7 (26.4–34.3)	29.7 (26.4–34.3)	29.6 (26.1–35.6)	0.713
MCV (fl)	86.7 (83.0.90.3)	88.0 (83.3–91.5)	85.8 (82.9–90.0)	0.450
WBC (×1000/μl)	7.4 (5.8–9.2)	7.1 (5.8–8.6)	8.2 (5.6–10.3)	0.095
Neutrophil (%)	73.0 (67.0–78.7)	72.0 (66.7–77.0)	77.0 (67.0–81.0)	0.109
Lymphocyte (%)	19.4 (13.9–26.0)	20.0 (14.9–27.0)	16.5 (11.9–23.0)	0.065
Absolute Neutrophil Count	5200.0 (3911.0–7101.7)	5040.0 (3870.0–6769.9)	5967.0 (3936.0–8287.9)	0.190
Absolute Lymphocyte Count	1452.0 (1008.8–1771.2)	1466.8 (1002.2–1948.5)	1389.2 (1045.2–1617.6)	0.199
Plt (/cumm)	220.0 (173.5–266.0)	230.5 (176.0–264.5)	206. (168.5–277.0)	0.413
NLR	3.7 (2.6–5.8)	3.5 (2.5–5.1)	4.6 (2.9–6.6)	**0.043** *
PLR	155.8 (126.0–220.8)	153.4(125.8–226.8)	162.5(126.0–213.8)	0.846
SII	768.0 (566.7–1247.9)	753.5 (533.5–1097.9)	866.8 (583.4–1389.1)	0.490
VBG-PH	7.3 (7.2–7.3)	7.3 (7.2–7.3)	7.3 (7.2–7.3)	0.427
VBG- HCO3 (mmol/L)	18.8 (15.7–22.7)	18.8 (16.1–22.6)	18.8 (15.7–22.7)	0.969
VBG-CO2 (mmol/L)	35.0 (28.7–40.0)	36.0 (31.0–40.4)	32.1 (27.9–39.8)	0.214
ESR (mm)	36.5 (19.7–56.5)	43.0 (25.0–61.0)	28.0(17.0–55.0)	0.267
Uric acid (mg/dl)	7.3 (5.8–8.8)	7.2 (5.9–8.8)	7.8 (5.3–9.0)	0.893
PTH (pg/ml)	147.0 (92.2–226.0)	158.0 (79.0–286.5)	131.0 (106.0–185.0)	0.911
Serum Alb (gr/dl)	3.5 (3.0–3.7)	3.6 (3.1–3.8)	3.1 (2.8–3.6)	**0.012** *
Total protein (gr/dl)	5.9 (5.1–6.4)	5.9 (5.3–6.1)	5.4 (4.5–6.6)	0.442
CRP				
Positive	52.1%	41.3%	68.4%	0.067
Negative	47.9%	58.6%	31.5%	
Proteinuria in Random urine analysis				
Positive	84.0%	80.3%	90.9%	0.181
Negative	16%	19.7%	9.1%	

Abbreviations: DM, Diabetes Mellitus; HTN, Hypertension; IHD/HF, Ischemic heart disease/heart failure; KT, Kidney Transplant; CVA, Cerebral Vascular Accident; AF, Atrial Fibrillation; HR, Heart Rate; SBP, Systolic Blood Pressure; DBP, Diastolic Blood Pressure; Cr, Creatinine; GFR, Glomerular Filtration Rate; BUN, Blood Urea Nitrogen; Ca, Calcium; Mg, Magnesium; K, Potassium; Na, Sodium; P, Phosphorus; VitD_3_, 25-OH vitamin D3; Hb, Hemoglobin; Hct, Hematocrit; MCV, Mean corpuscular volume; WBC, White Blood Cell;; Plt, Platelets; NLR, Neutrophil-to-Lymphocyte Ratio; PLR, Platelet-to-Lymphocyte Ratio; SII, Systemic Immune Inflammation index; VBG, venous blood gas; ESR, Erythrocyte Sedimentation Rate; PTH, Parathyroid Hormone; Serum Alb, Serum Albumin; CRP, C-Reactive Protein

*Statistically significant

** The median and interquartile range (IQR) were reported for quantitative variables.

***Patients with uncontrolled hypothyroidism were excluded due to its potential link with pericardial effusion. The table displays individuals with a history of hypothyroidism and controlled TSH levels.

### Clinical and laboratory variables on admission and the date of echocardiography

The gender ratio was not statistically significant between groups. Clinically, chest pain and dyspnea were notably more prevalent in patients with moderate to severe PE at admission (p-value: 0.03 and 0.013 respectively), as detailed in Table 1. Laboratory findings revealed a higher NLR (4.6 vs. 3.5, p-value: 0.043) and lower serum albumin (3.1 vs. 3.6 g/dl, p-value: 0.012) in patients with moderate to severe PE compared to those with mild PE.

The average interval between admission and echocardiography was 3 days, ranging from 0 to 24 days. Analysis at the time of initial echocardiography showed significantly higher white blood cell count (WBC) (8.2 vs. 6.8 ×1000/μl, p-value: 0.008), neutrophil percentage (75.7% vs. 68.7%, p-value: 0.020), and count (6000 vs. 4408, p-value: 0.012), and NLR (4.6 vs 3, p-value: 0.012), along with a notably lower lymphocyte percentage (16.2% vs 23%, p-value: 0.004) and serum albumin (3 vs 3.5 g/dl, p-value: 0.023) in patients with moderate and severe PE, as shown in Table 2.

**Table 2:** Laboratory variables at the date of echocardiography.

Variable**	Total (n = 112)	Mild (n = 72)	Moderate & Severe (n = 40)	P-value
Cr (mg/dl)	4.1 (3.1–6.3)	4.3 (3.1–6.0)	3.9 (3.1–6.4)	**0.763**
GFR (ml/min)	11.5 (9.0–17.0)	11.0 (9.0–17.0)	12.0 (9.7–19.2)	**0.714**
BUN (mg/dl)	59.3 (43.0–73.0)	61.2 (44.8–80.8)	51.4 (40.1–64.0)	**0.095**
Ca (mg/dl)	8.4 (7.8–8.9)	8.5 (8.0–9.0)	8.4 (7.6–8.7)	**0.228**
Corrected Ca (mg/dl)	8.9 (8.2–9.4)	8.9 (8.3–9.4)	8.9 (8.2–9.4)	**0.820**
Mg (mg/dl)	2.2 (1.9–2.5)	2.2 (2.0–2.5)	2.2 (1.9–2.5)	**0.989**
K (mEq/L)	4.6 (4.1–5.0)	4.6 (4.1–5.0)	4.6 (4.1–5.0)	**0.560**
Na (mEq/L)	139.0 (136.0–142.5)	141.0 (137.0–143.0)	138.5 (135.0–142.0)	**0.232**
P (mg/dl)	5.1 (4.3–6.5)	5.1 (4.4–6.2)	5.1 (4.0–6.6)	**0.918**
Hb (gr/dl)	9.1 (8.1–10.6)	9.4 (8.2–10.5)	8.8 (7.8–10.9)	**0.882**
Hct (%)	29.7 (26.2–34.6)	30.0 (26.4–34.6)	29.1 (25.4–35.2)	**0.996**
WBC (×1000/μl)	7.4 (5.1–9.1)	6.8 (4.9–8.4)	8.2 (5.6–10.7)	0.008*
Neutrophil (%)	70.9 (64.2–79.0)	68.7 (63.1–75.0)	75.7 (65.9–80.9)	0.020*
Lymphocyte (%)	20.6 (14.8–27.6)	23.0 (17.5–29.5)	16.2 (12.9–23.0)	0.004*
Absolute Neutrophil Count	4806.0 (3431.0–6806.0)	4408.0 (3327.1–6014.0)	6000.0 (3790.5–8378.1)	0.012*
Absolute Lymphocyte Count	1484.2 (1074.6–1863.9)	1498.0 (1100.2–1955.0)	1482.4 (1042.6–1656.2)	**0.315**
Plt (/cumm)	212.0 (170.0–245.2)	215.5 (170.7–243.2)	206.0 (154.2–278.5)	**0.902**
NLR	3.4 (2.3–5.4)	3.0 (2.2–4.4)	4.6 (2.8–6.1)	0.012*
PLR	140.0 (110.0–201.4)	138.5 (107.9–198.9)	147.8 (116.0–207.6)	**0.803**
SII	705.7 (459.6–1036.6)	675.7 (434.6-903/1)	750.3 (526.5–1342)	**0.176**
VBG-PH	7.3 (7.2–7.3)	7.3 (7.2–7.4)	7.3 (7.2–7.3)	**0.934**
VBG-HCO3 (mmol/L)	20.3 (16.9–23.3)	20.7 (17.3–23.6)	19.2 (16.0–22.7)	**0.421**
VBG-CO2 (mmol/L)	35.0 (28.6–40.9)	37.0 (31.6–41.7)	32.0 (27.5–39.6)	**0.092**
ESR (mm)	33.0 (19.7–67.5)	43.0 (23.7–67.5)	28.5 (17.2–70.0)	**0.487**
Uric acid (mg/dl)	7.4 (6.0–8.5)	7.3 (5.9–8.4)	8.0 (6.4–8.8)	**0.545**
Serum Alb (gr/dl)	3.4 (3.0–3.7)	3.5 (3.0–3.8)	3.0 (2.8–3.5)	0.023*
Total protein (gr/dl)	5.9 (5.3–6.4)	5.9 (5.3–6.4)	5.5 (4.9–6.6)	**0.725**

*Statistically significant

** The median and interquartile range (IQR) were reported for quantitative variables.

Abbreviations: Cr, Creatinine; GFR, Glomerular Filtration Rate; BUN, Blood Urea Nitrogen; Ca, Calcium; Mg, Magnesium; K, Potassium; Na, Sodium; P, Phosphorus; VitD3, 25-OH vitamin D3, Hb, Hemoglobin; Hct, Hematocrit; MCV, Mean corpuscular volume; WBC, White Blood Cell; Plt, Platelets; NLR, Neutrophil-to-Lymphocyte Ratio; PLR, Platelet-to-Lymphocyte Ratio; SII, Systemic Immune Inflammation Index; VBG, venous blood gas; ESR, Erythrocyte Sedimentation Rate; PTH, Parathyroid Hormone; Serum Alb, Serum Albumin.

### Dialysis initiation and pericardial effusion drainage

Among the 112 patients studied, 53 underwent hemodialysis during their hospital stay. There was a significant association between the severity of PE and the initiation of dialysis for any clinical reason (odds ratio (OR): 2.22, 95% Confidence Interval (CI): 1.011–4.9, p-value: 0.045) as shown in Table 3. Pericardial fluid drainage was performed in 21 patients, with 16 experiencing severe and 5 experiencing moderate PE. The composition of the drained pericardial fluid varied, being bloody in 47.1%, serous in 35.3%, and serosanguineous in 17.6% of cases, detailed in Table 4. The need for pericardial fluid drainage was significantly associated with the severity of PE (p-value < 0.001), as indicated in Table 5.

**Table 3:** Patients that underwent dialysis during the time of admission due to any indication (before or after the diagnosis of pericardial effusion).

PE Severity	Total (n = 112)	Treatment with dialysis		OR (95%CI)	P-value
		No (n = 59)	Yes (n = 53)		
**Moderate and Severe PE**	40 (35.7%)	16 (27.1%)	24 (45.3%)	2.22 (1.011–4.9)	0.045*
**Mild (reference)**	72 (64.3%)	43 (72.9%)	29 (54.7%)	-	-

*Statistically significant

Abbreviations: PE, pericardial effusion

**Table 4:** Pericardial fluid characteristics of the patients who have undergone pericardial drainage (n = 21).

Degree of pericardial effusion in echocardiography	Severe (n = 16); Moderate (n = 5); Mild (n = 0)
**Amount of fluid (milliliters)**	Mean = 841; Std. Error of Mean = 118.7; SD = 490; Median = 750, IQR: 550–1000; Range: 300–2000
**Type of effusion**	Bloody = 47.1%; Serous = 35.3%; Serosangious = 17.6%

**Table 5:** The association between pericardial effusion severity and pericardial fluid drainage.

**PE Severity**	**Pericardial fluid drainage**			**P-value**	**Likelihood Ratio**
	Total (n = 112)	No (n = 91)	Yes (n = 21)		
Moderate and severe PE	40 (35.7%)	19 (20.9%)	21 (100.0%)	**0.000** *	52.74
Mild (reference)	72 (64.3%)	72 (79.1%)	0 (0.0%)		
**PE Severity**	**Pericardial fluid drainage**			**P-value**	**Likelihood Ratio**
	Total (n = 40)	No (n = 19)	Yes (n = 21)		
Severe PE	16 (40.0%)	0 (0.0%)	16 (76.2%)	**0.000** *	30.79
Moderate (reference)	24 (60.0%)	19 (100.0%)	5 (23.8%)		

*Statistically significant

Abbreviations: PE, pericardial effusion

### Mortality

Our study found no significant correlation between PE severity and mortality rates during the current hospital admission (p-value: 0.613).

### Predictors of pericardial effusion severity in CKD patients

Multivariable binary logistic regression analysis, based on admission data, indicated patients with hypoalbuminemia (serum albumin < 3.5 g/dl) (OR: 4.03, p-value 0.014) and NLR level greater than 5.5 (OR: 4.22, p-value 0.015) are more likely to have moderate and severe PE (Table 6). Furthermore, the analysis at the time of echocardiography revealed significant associations between serum albumin and neutrophil count with PE severity; patients with hypoalbuminemia (OR: 5.38, p-value 0.004) and neutrophilia (OR: 7.94, p-value: 0.005) were more likely to experience moderate to severe PE (Table 6).

**Table 6:** Comparison of laboratory indexes at admission and echocardiography time between pericardial effusion groups: Multivariable logistic regression analysis.

**admission time**	**Variable**	**cut-off**	**Crude OR (95%CI)**	**P-value**	**Adjusted OR (95%CI)**	**P-value**
	Alb^a^	<3.5	2.42 (0.96–6.10)	0.061	4.03 (1.32–12.25)	0.014*
	NLR^a^	>5.5	4.05 (1.57–10.48)	0.004	4.22 (1.32–13.50)	0.015*
**Echocardiography time**	Alb^a^	<3.5	3.46(1.32–9.01)	0.011	5.38(1.74–16.65)	0.004*
	NLR^a^	>5.5	3.47(1.24–9.69)	0.017	-	-
	ANC^c^	>7700	7.38(2.20–24.78)	0.001	7.94(1.89–33.44)	0.005*

^a^Serum albumin ≥3.5 (gr/dl) considered as a reference

^b^NLR≤ 5.5 considered as a reference

^c^ANC≤7700 considered as a reference

*Statistically significant

Abbreviations: Alb, Serum albumin; NLR, Neutrophil-to-Lymphocyte Ratio; ANC, Absolute Neutrophil Count

## Discussion

Our study highlights dyspnea, chest pain, serum albumin, and an NLR level exceeding 5.5 upon admission, along with hypoalbuminemia and neutrophilia at the time of echocardiography, as significant predictors of moderate to severe PE in CKD patients.

Pericardial involvement in late-stage CKD, often manifesting as pericarditis or PE, is a notable complication. While initially uremic pericarditis was thought to occur without substantial effusion, later research indicates a high frequency of asymptomatic PE in these patients, with 70–100% of those with uremic and dialysis-associated pericarditis experiencing PE [4, 30].

Many CKD-related PE cases initially show no symptoms, even with significant effusion, posing a challenge for early diagnosis [31, 32]. Our findings corroborate this, showing that a majority of PE patients did not report chest or epigastric discomfort. Specifically, 81% of patients with PE and 60.8% with moderate to severe PE were asymptomatic in these regards. Despite not being a definitive symptom, dyspnea was noted in 54% of PE patients, increasing to 71.4% in moderate to severe cases. Furthermore, 70.7% showed peripheral edema, indicative of volume overload [33].

This finding is consistent with the observations made by Frommer et al., who reported clinical and radiological signs of volume overload in a group of 50 non-dialysis ESRD patients with PE. In their study, 36% of the patients had asymptomatic PE, and 6% displayed clinical features of pericarditis without PE [6]. Building upon our earlier observation that PE, even large ones, often remains asymptomatic in CKD patients, it’s crucial to consider the implications of undiagnosed PE, particularly in those requiring dialysis. In such cases, if PE goes unrecognized, the typical high ultrafiltration rate in dialysis can result in decreased central venous pressure and venous return. This scenario can lead to hypotension, a serious complication [28, 34]. Therefore, heightened clinical vigilance for CKD-related PE and knowledge of its predictors are essential in preventing these severe outcomes [7].

In evaluating our findings in relation to existing literature, it is important to consider the variances in methodologies and study populations. Prior research in this field displays a wide range of participant demographics, including individuals on dialysis, those not on dialysis, and mixed groups. Furthermore, the comparative analyses in these studies vary greatly, from comparing patients with and without PE to examining PE groups of different severity levels. The variation may also arises from differences in reporting timeframes, definitions of PE and its severity grades, and disparities in diagnostic and treatment methodologies. This diversity necessitates a nuanced approach when comparing results across different studies.

In Leehay’s study of 47 ESRD patients (11 patients had not undergone dialysis), significant differences were noted in dyspnea, tachypnea, low voltage ECG, pleural effusion presence, and left shift in WBCs between patients with PE exceeding 250 cc and those with less than 250 cc [38]. Frommer et al., found a higher incidence of PE in diabetics (58.3%) compared to non-diabetics (29%), with lower serum albumin in diabetics. However, the small sample size was a limitation [9]. In the case-control study by Ravi et al., involving 84 stage 4 and 5 CKD patients with PE, 44% were receiving dialysis. Notably, 47% of these patients exhibited moderate to large PE. The study identified higher heart rate (OR: 1.290 per 10bpm), elevated potassium level (OR: 1.949 per 1‐mEq/L), and lower corrected calcium (OR: 1.33 per 1‐mg/dL) as independent predictors for the presence of any PE. A specific finding was that a corrected calcium level below 8 mg/dL showed a high specificity for moderate and large PE compared to those with no effusion. Furthermore, in patients not on dialysis, corrected calcium was the sole significant predictor for PE. Notably their study, did not find an association between PE and a worse short-term prognosis [31].

In a study involving 2820 outpatient ESRD patients, 79.5% were undergoing dialysis at the time of echocardiography. Among these, 54 exhibited moderate to large PE which 75.9% had moderate PE, 24.1% had large PE, and 13% experienced tamponade and 70.4% of these 54 patients received intermittent dialysis. The study evaluated clinical and laboratory factors, including serum albumin and calcium, comparing PE patients with those without PE in a 1:2 ratio. The findings suggested that the duration of hemodialysis was a key protective factor against developing moderate to large PE. Patients in this study were followed for an average of 39 months, revealing a 10-year survival rate of 87%, indicating a favorable prognosis [35].

In the study by Yoshida et al., echocardiography was performed on 150 patients with chronic uremia, both before and during hemodialysis treatment. The study revealed significant difference in systolic blood pressure, dilation of the left atrial chamber, anemia, and hypoproteinemia between patients with and without PE. However, no notable differences were observed in creatinine, uric acid, calcium, or body weight changes across patients with varying degrees of PE [10].

In CKD patients at earlier stages, PE tends to arise from factors unrelated to renal disease. Interestingly, in late-stage CKD patients, we did not observe any significant association between serum creatinine, GFR, and BUN with the severity of PE. Previous studies have similarly reported a lack of correlation between these parameters and the presence or severity of PE [8, 10, 31]. However, Matsumoto et al.’s study in 131 uremic patients, observed that among 65 patients who did not undergo dialysis, 25 of them displayed PE and serum creatinine levels exceeding 5 mg/100 ml. The authors noted an increased likelihood of uremic PE in correlation with elevated creatinine levels within this subgroup [36]. Additionally, Frommer et al. noted in pre-dialysis ESRD patients that those with PE tended to have lower BUN levels [9].

It’s important to highlight that the NLR and platelet-to-lymphocyte ratio (PLR) have recently been recognized as innovative inflammatory markers associated with adverse outcomes in a range of medical conditions [37]. However, the exploration of their relationship with PE in CKD patients has not been extensively pursued in existing research. This gap suggests a potential area for future studies to investigate the role of these markers in the context of PE among CKD patients PE, particularly in moderate to severe cases, carries the risk of progressing to tamponade. This risk is significant, with about one-third of severe PE cases, regardless of cause, evolving into tamponade. It’s crucial to note that occurrence of tamponade isn’t solely contingent on the volume of PE; the rate of fluid accumulation within the pericardial space also plays a pivotal role [2]. Our current study established that the severity of PE can serve as a predictor for the necessity of surgical intervention. None of the patients with mild PE required drainage, whereas all with severe PE and about one-fifth of those with moderate effusion underwent this procedure, aligning with previous research that associates the extent of PE with an increased likelihood of surgical intervention [38].

In Bataille et al.’s study of 44 CKD patients with PE, 43% were undergoing hemodialysis, 7% were on peritoneal dialysis, 11% had undergone transplantation, and 39% were at CKD stage 4 or 5. They reported that severe effusion often necessitated surgical drainage, as did 35% of mild to moderate effusion cases with serum albumin below 3.1 g/dl. Only 7% of patients with mild to moderate effusion and albumin levels above 3.1 g/l underwent drainage. The study concluded that large effusions in CKD patients should be promptly drained. For mild to moderate effusions, serum albumin levels can guide the decision for drainage. However, the study didn’t specify if there were changes in effusion volume between initial diagnosis and drainage, despite potential delays up to 136 days [39]. Although the risk of drainage in PE is influenced by its severity, directly comparing our results with their study is challenging due to differences in population groups, methodologies, and data reporting. Additionally, some studies report no significant relationship between serum albumin and PE in mixed populations of dialysis and non-dialysis CKD patients [31, 35], further illustrating the variability in findings across different research contexts.

Our study did not find a significant relationship between the severity of PE and in-hospital mortality, aligning with previous studies that suggest a favorable short-term prognosis for CKD patients with PE who receive prompt management [31, 35]. Further details on the review of related studies can be found in S3 Table.

### Limitations

Our study’s reliance on admission creatinine levels for CKD staging, due to the absence of baseline data, is a limitation, as it might not accurately reflect chronic kidney function in cases of acute kidney injury superimposed on CKD. Additionally, the potential for PE causes other than CKD in some patients could not be definitively excluded. The retrospective design, based on hospital record analysis, may introduce certain biases. Future larger-scale, prospective studies are needed to further validate and strengthen our findings.

## Conclusions

Despite advancements in diagnosing and treating CKD, PE remains a significant concern for CKD patients. We have identified hypoalbuminemia, neutrophilia, and NLR as predictive factors for moderate and severe PE in CKD patients with PE. These findings can aid physicians in recognizing and addressing moderate to large PE in this population. However, prospective studies with larger sample size are necessary to validate our results.

## Supporting information

S1 Table Patients registered with chronic kidney diseases (before eight weeks of dialysis initiation) in whom the presence of pericardial effusion could be explainable by another medical condition. (DOCX)

S2 Table Patients registered with moderate to severe pericardial effusion and chronic kidney diseases stages 4 and 5 (before eight weeks of dialysis) in whom the presence of pericardial effusion could be explainable by another medical condition. (DOCX)

S3 Table Review of the most related literature. (DOCX)
