# Supplementary material for: Pericardial effusion in patients with chronic kidney disease: A two-center study
Source: PLoS One. 2024 Jun 6;19(6):e0302200. doi: 10.1371/journal.pone.0302200 (PMC11156368; doi:10.1371/journal.pone.0302200)
Supplement: S1 Table — (DOCX) [file pone.0302200.s001.docx]

**S1 Table.** Patients registered with chronic kidney diseases (before eight weeks of dialysis initiation) in whom the presence of pericardial effusion could be explainable by another medical condition.

| Total number | **Subtype** | | **Cause of pericardial effusion** |
| --- | --- | --- | --- |
| 35 |  |  | **Infections** |
|  | 4 | SARS-COV-2 |  |
|  | 1 | HSV |  |
|  | 1 | CMV |  |
|  | 1 | TB |  |
|  | 10 | positive blood culture and Sepsis |  |
|  | 11 | Pneumonia |  |
|  | 1 | Endocarditis |  |
|  | 6 | Others |  |
| 4 |  |  | **Autoimmune and auto-inflammatory** |
|  | 1 | Lupus |  |
|  | 1 | Rheumatoid arthritis |  |
|  | 1 | FMF |  |
|  | 1 | Granulomatous with polyangiitis |  |
| 6 |  |  | **Neoplasms** |
|  | 1 | Leukemia |  |
|  | 2 | Multiple myeloma |  |
|  | 3 | Genitourinary cancer |  |
| 8 |  |  | **Cardiac** |
|  | 5 | Post cardiac surgery |  |
|  | 3 | Post myocardial infarction |  |
| 4 |  |  | **Metabolic** |
|  | 4 | Hypothyroidism) uncontrolled( |  |
| 3 |  |  | **Drugs** |
|  | 3 | Hydralazine |  |
|  | 0 |  | **Radiation** |
|  | 0 |  | **Trauma** |
|  |  |  | **Idiopathic** |
| 60 |  |  | **Total** |
