# Supplementary material for: Pericardial effusion in patients with chronic kidney disease: A two-center study
Source: PLoS One. 2024 Jun 6;19(6):e0302200. doi: 10.1371/journal.pone.0302200 (PMC11156368; doi:10.1371/journal.pone.0302200)
Supplement: S2 Table — (DOCX) [file pone.0302200.s002.docx]

**S2 Table.** Patients registered with moderate to severe pericardial effusion and chronic kidney diseases stages 4 and 5 (before eight weeks of dialysis) in whom the presence of pericardial effusion could be explainable by another medical condition.

| Total number | | **Subtype** | **Cause of pericardial effusion** |
| --- | --- | --- | --- |
| 12 |  |  | **Infections** |
|  | 1 | SARS-COV-2 |  |
|  | 1 | Influenza |  |
|  | 4 | TB |  |
|  | 1 | Positive pericardial culture |  |
|  | 1 | Toxic shock syndrome |  |
|  | 3 | Pneumonia |  |
|  | 1 | Pyelonephritis |  |
| 7 |  |  | **Autoimmune and auto-inflammatory** |
|  | 5 | Lupus |  |
|  | 1 | Rheumatoid arthritis |  |
|  | 1 | Scleroderma |  |
| 10 |  |  | **Neoplasms** |
|  | 3 | Lung cancer |  |
|  | 1 | Mediastinal tumor |  |
|  | 2 | Breast cancer |  |
|  | 1 | Hematologic cancer |  |
|  | 2 | Gastrointestinal cancer |  |
|  | 1 | Skin cancer with metastasis |  |
| 6 |  |  | **Cardiac** |
|  | 4 | Post cardiac surgery |  |
|  | 1 | Post mi |  |
|  | 1 | Aortic intramural hematoma |  |
| 2 |  |  | **Metabolic** |
| 0 |  | Hypothyroidism |  |
| 0 |  |  | **Drugs** |
| 0 |  |  | **Radiation** |
| 0 |  |  | **Trauma** |
| 0 |  |  | **Idiopathic** |
| 37 |  |  | Total |
