# Supplementary material for: Pericardial effusion in patients with chronic kidney disease: A two-center study
Source: PLoS One. 2024 Jun 6;19(6):e0302200. doi: 10.1371/journal.pone.0302200 (PMC11156368; doi:10.1371/journal.pone.0302200)
Supplement: S3 Table — (DOCX) [file pone.0302200.s003.docx]

| **S3 Table. Review of the most related literature.** | | | |
| --- | --- | --- | --- |
| **Main finding/ outcome** | **The population of the study** | **Year** | **Authors** |
| Pioneered the description of pericarditis in the necropsy reports of 8 out of 100 patients afflicted with uremia. | Hundred patients with Uremia | 1836 | Richard bright et al. |
| Observed pericardial effusion in post-mortem examinations of patients with chronic uremia. | Chronic uremia | 1922 | Barach et al. |
| Presented a case of hemopericardium emerged from the rupture of the right coronary artery in a patient with chronic interstitial nephritis. | Case report | 1922 | Jones-Evans et al. (1) |
| They found pericardial effusion in the autopsy of patients with chronic uremia. | Chronic uremia | 1936 | Richter and O'Hare |
| Outlined electrocardiographic alterations attributed to hyperkalemia in three chronic/acute uremic patients. Noted pericardial effusion/uremic pericarditis in the autopsy of two cases, notwithstanding the presence of additional medical conditions potentially associated with pericardial effusion. | patients with uremia /Case series | 1944 | Keith et al.(2) |
| Normal pericardium in the necopsy of 13/ 27 patients.  Serous or hemorrhagic pericardium in 5 /27 cases.  Acute fibrous pericarditis 7/27 | Twenty-seven Patients with Chronic uremia | 1949 | Langendrof and pirani (1, 3) |
| Noted a significant presence of serous or hemorrhagic effusion in patients with uremic pericarditis. | Uremic pericarditis | 1954 | Fish-berg et al. (4) |
| Documented two cases of cardiac tamponade in non-dialyzed patients with uremic pericarditis, both of them died.. | Case report | 1956 | Goodner and brown (5, 6) |
| Two instances of cardiac tamponade due to chronic uremia were documented in non-dialyzed patients. In both cases, timely diagnosis and pericardial fluid drainage led to improvement. Unfortunately, hyperkalemia proved fatal in one case. | Case report | 1957 | Guild et al.(1, 6) |
| Among 227 cases of uremic pericarditis, 58 exhibited pericardial effusion,  and 3 had massive hemorrhagic effusion | Two hundred twenty-seven patients with uremic pericarditis | 1960 | Lowry and Boyd (4) |
| Reported a case of cardiac tamponade in a chronically uremic patient, highlighting the importance of timely intervention for full recovery. | Case report | 1962 | Merikas G et al (7) |
| Three patients underwent hemodialysis within four weeks preceding tamponade onset, while one received no prior dialysis. Underlying uremia causes included chronic glomeronephritis, amyloidosis, acute tubular necrosis, and prostatic hypertrophy with chronic pyelonephritis. Although no direct correlation between blood urea levels and tamponade was observed, hemodialysis may potentially influence this relationship. | Four cases of cardiac tamponade due to uremic pericarditis/ Case series | 1964 | [Symons](https://pubmed.ncbi.nlm.nih.gov/?term=Symons%20HS%5BAuthor%5D) and Wrong (4) |
| All admitted patients exhibited severe uremia and received dialysis. The diagnosis of pericarditis was made after dialysis initiation in 11 cases, with the majority occurring within two months.  Cardiac tamponade occurred in 15 patients, with only two cases experiencing it prior to dialysis. Typically, tamponade manifested 2-3 days after hemodialysis initiation.  Survival post-tamponade diagnosis ranged from 0 to 19 months, and notably, 10 patients survived beyond 4 months.  It is worth mentioning that electrocardiographic evidence of pericarditis was evident in only 6 of the 25 patients. Additionally, the significant incidence of tamponade post-hemodialysis raised questions about whether heparin played a role in inducing pericardial effusion in these patients. | Twenty-seven patients with the diagnosis of pericarditis due to uremia. | 1966 | Beaudry et al. (8) |
| The study employed angiocardiography and cholangiogram I scans for the detection of pericardial effusion, with diagnostic confirmation achieved through pericardiocentesis/ autopsy.  Comparative analysis of laboratory findings was conducted in conjunction with effusion-free intervals. Among thse cases, eleven patients had chronic kidney disease, while one exhibited acute tubular necrosis.  Notably, four individuals did not receive dialysis prior to the diagnosis of pericardial effusion. Additionally, five patients underwent dialysis on fewer than ten occasions before the onset of pericardial effusion. Two patients underwent peritoneal dialysis proceeding with the effusion and one of these patients having undergone dialysis six months prior to the diagnosis.  The determination of the hemodynamic significance of pericardial effusion was challenged by the concomitant presence of congestive heart failure attributed to volume overload. Alterations in urinary sodium and chloride excretion and reduction in cardiac output, emerged as the most salient factors associated with the occurrence of cardiac tamponade.  The study further demonstrated notable changes in the composition of pericardial fluid attributable to dialysis, although an increase in pericardial volume was not observed as a direct consequence of the procedure.  Moreover, the research reported a noteworthy survival rate of 83% during the follow-up period, which extended to approximately 19 months. | 12 patients with uremic hemopericardium | 1968 | Allen et al.  (9) |
| Uremia-induced pericarditis emerged as one of the prominent causative factor for pericardial effusion. | Patients with pericardial effusion | 1976 | Krayenbühl,et al. (10) |
| The predominant etiological factor for pericardial effusion in 31 out of 46 cases (67%) was identified as uremia. | Forty-six patients with pericardial effusion were treated by subxiphoid approach. | 1977 | Gil et al.(11) |
| Echocardiography revealed pericardial effusion in 33 patients, categorized as mild (21), moderate (9), and severe (1). | Fifty patients with chronic renal failure, including those on long-term dialysis, presented with symptoms in favor of cardiac disease. | 1978 | D'Cruz et al.(12) |
| Sixty-one of these patients had pericardial effusion during the time of the study.  The classification included: 40 patients with solely uremia, 14 with uremia complicated by infection, and 7 presenting uremia alongside other concurrent conditions.  Cardiac tamponade was confirmed in 7 cases, and 16 of the 61 patients succumbed during the study period. Pericardial effusion within the uremic group typically exhibiting an asymptomatic course. Notably, this group often responded positively to dialysis alone, whereas the remaining two cohorts demonstrated limited responsiveness to dialysis.  Among the 56 patients receiving dialysis, 36 were diagnosed with pericardial effusion, while 25 out of the 65 patients who did not undergo dialysis exhibited this condition. It is noteworthy that these 25 patients exhibited Creatinine levels surpassing 5 mg/100 ml; the authors posit an increased likelihood of uremic pericardial effusion with elevated creatinine levels in this group.  Ultimately, 48 out of the 65 patients initiated dialysis during the study period.  They suggest that echocardiography should be done routinely in patients under dialysis. | One hundred thirty-one uremic patients (including those treated with dialysis) | 1980 | Matsumoto et al.(13) |
| In their study of 150 patients with chronic uremia, echocardiography was conducted both before and during hemodialysis treatment. The reported incidence of pericardial effusion was 62%, notably higher within the initial three months of dialysis. Patients were categorized based on pericardial effusion severity (graded 0-5). The study revealed significant disparities in systolic blood pressure, left atrial chamber dilatation, anemia, and hypoproteinemia between patients with and without effusion. However, no substantial differences were observed in creatinine levels, uric acid, calcium, or changes in body weight across patients with varying degrees of pericardial effusion. | One hundred fifty patients with chronic uremia. | 1982 | Yoshida et al. (14) |
| Among 25 patients undergoing subxiphoid pericardial window procedures, uremia was identified as the cause of pericardial effusion in 12 cases. Encouragingly, eleven of these patients remain alive and free from recurrence, with postoperative durations ranging from 3 to 36 months. | Twenty-five patients that underwent subxiphoid pericardial window | 1982 | Prager et al.(15) |
| Of the total cohort, 18 out of 50 (36%) exhibited asymptomatic pericardial effusion of varying degrees. While three patients displayed clinical features of pericarditis, none manifested pericardial effusion. Clinical symptoms and signs of overload, including dyspnea, orthopnea, paroxysmal nocturnal dyspnea, the third heart sound, pulmonary rales, and jugular venous distention, along with radiological indicators of overload, were significantly more pronounced in patients with pericardial effusion. Additionally, blood urea nitrogen levels were notably lower in this subgroup. Noteworthy, among the 50 patients, 12 had a history of diabetes mellitus, of which 7 had concurrent pericardial effusion (58.3%). In comparison, among the 38 patients without diabetes, eleven (29%) developed pericardial effusion. This discrepancy proved statistically significant. Furthermore, diabetic patients exhibited significantly lower serum albumin levels compared to the non-diabetic population. The study also tracked 33 out of the 50 patients following the initiation of chronic dialysis, with no new cases of pericardial effusion reported. Changes in effusion size were found to correlate with alterations in body weight between dialysis sessions. | Fifty patients with the end-stage renal disease just before the treatment with chronic dialysis | 1985 | Frommer et al. (16) |
| In 6/ 32 patients, uremia was the cause of the pericardial effusion. | Thirty-two patients with pericardial effusion | 1984 | Little et al.(17) |
| Eleven of them had not undergone prior dialysis. Among these, three patients had pericardial effusions exceeding 250 cc, with two eventually requiring surgical drainage. Among the total of 47 patients, effusion size emerged as the most potent predictor for surgical intervention. Additionally, in cases where this factor was not considered, a left shift in WBC count and tachypnea also demonstrated predictive value for the necessity of surgery. The study compared patients with effusions larger than 250 cc to those with smaller volumes. In the multivariate regression analysis, associations were found with low voltage in ECG, pleural effusion, and a somewhat lower yet significant association with the left shift in WBC count. | Forty-seven patients with ESRD who had pericardial effusion | 1989 | Leehey et al. (18) |
| The cause of effusion in 9 of these 41 patients was uremia. | Forty-one patients that underwent subxiphoid pericardial tube drainage | 1989 | Palatianos et al. (19) |
| Out of 50 patients, 15 presented with pericardial effusion. | Fifty patients with chronic renal failure (42 male, 8 female) | 1989 | Achari et al.(20) |
| Uremia was identified as the cause of significant pericardial effusion in 7 out of 57 patients. | Fifty-seven patients with large pericardial effusion had undergone subxiphoid pericardiotomy. | 1992 | Wall et al. (21) |
| Among the 22 patients who underwent the procedure, uremia was established as the cause of pericardial effusion in 7 cases. | Twenty-two patients underwent thoracoscopic partial pericardiectomy. | 1997 | Robles et al. (22) |
| The etiology of pericardial effusion in 3 of these patients was reported as chronic renal failure. | seventy-eight Patients who had undergone echo-guided pericardiocentesis in the intensive care unit in 3 different hospitals. | 1998 | Vayre, et al. (23) |
| The etiology of pericardial effusion was attributed to uremia in 12 out of 70 patients who underwent pericardial drainage. In their study, tuberculosis emerged as the most prevalent etiological factor for pericardial effusion, affecting 24 out of 70 cases. | Seventy patients that undergone pericardial drainage | 1999 | Dogan et al. (24) |
| Among these, four out of the 63 diagnosed with cardiac tamponade were ESRD patients, with three developing cardiac tamponade either prior to or within the initial two months of dialysis initiation. Notably, none of these patients experienced a recurrence of cardiac tamponade. | Sixty-three patients with cardiac tamponade underwent pericardiocentesis in the intensive care unit | 2000 | Bastian et al. (25) |
| 55% had severe PE, 41% moderate, and 4% mild. Uremia was the cause in 42% of cases. Among 101 uremic patients, there was one fatality within 30 days. Sixteen had recurrence, leading to further intervention. Patients were monitored for at least a year; only one uremic patient passed away, within the first 30 days. | Two hundred-forty patients with pericardial effusion, who underwent subxiphoid pericardiostomy | 2003 | Becit et al. (26) |
| They attributed uremia as the cause of pericardial effusion if the Cr level exceeded 2 mg/dl, or if patients had nephrotic syndrome, or were under dialysis. According to their criteria, ten patients (6%) were diagnosed with uremic pericardial effusion. However If a patient exhibited these criteria along with another potential cause, they were classified as having indeterminate pericardial effusion. | One hundred seventy-three patients that undergone pericardiocentesis. | 2006 | Ben-Horin, et al. (27) |
| Notably, 63.2% of cases were classified as mild pericardial effusion. The leading identified causes of pericardial effusion were chronic renal failure, accounting for 25% of admissions with any level of pericardial effusion, followed by malignancies. The predominant clinical symptom was dyspnea. | One hundred thirty-six patients with the diagnosis of pericardial effusion. | 2010 | Gümrükçüoğlu, et al. (28) |
| Among the patients on conservative treatment, 14 (22%) exhibited pericardial effusion. For those undergoing regular hemodialysis, 12 (19%) displayed pericardial effusion. Additionally, 7 (11%) had minimal effusion, while 31 (48%) did not have any pericardial effusion. In the subset of CKD patients with minimal effusion, seven (11%) had this condition, five (8%) presented minimal pericardial effusion, and the remaining two (3%) showed no signs of pericardial fluid accumulation. - Regarding the size of pericardial effusion, it was categorized as follows: Small: 17 (27%), Moderate: 9 (14%), and Large: 4 (6%). Notably, four patients exhibited a thickened pericardium, of which three did not have pericardial effusion, and one had minimal effusion. - The reported prevalence of pericardial effusion in CKD patients, inclusive of those on regular hemodialysis, was approximately 48%. - The study's conclusion is that the presence of a positive Echo-Free Space (EFS) without accompanying clinical symptoms does not definitively confirm the presence of pericarditis in CKD patients. | Sixty-four patients with CKD. | 2011 | Zahiti, et al. (29) |
| Patient Distribution:  Hemodialysis: 43% - Peritoneal Dialysis: 7% - Transplanted: 11% - CKD Stages 4 and 5: 39%  Etiologies: - Uremic Pericarditis: 45.4% - Dialysis  associated Pericarditis: 45.5% - Other: 9%  Pericardial Effusion Size:  Small: 30% - Moderate: 32% - Large: 30%   Treatment : All patients with large effusion eventually required drainage. For small and moderate effusion, 35% with serum albumin below 31g/l underwent drainage, compared to only 7% with levels above 31g/l.  Time between diagnosis and drainage varied, with a maximum delay of 136 days, Five patients with small PE in the first echocardiography and 2 patients with a moderate amount of effusion eventually underwent drainage. Effusion changes at drainage were not reported.  Conclusion:  Large effusion necessitates prompt drainage. Serum albumin levels can help predict the need for drainage in cases of small to moderate effusion.   One patient in the study, with dialysis-associated pericarditis, passed away due to tamponade. Clinical signs were absent, but echocardiography indicated cardiac tamponade. | Forty-four CKD patients with pericardial effusion. | 2015 | Bataille, et al.  (30) |
| Out of 251 patients, 137 were on intermittent hemodialysis, and 114 received emergent hemodialysis. Among them, 89 had varying degrees of pericardial effusion. Out of the 114 who had emergent hemodialysis, 51 had pericardial effusion, including 7 with severe cases and 4 with tamponade. Among the 137 on intermittent hemodialysis, 38 had pericardial effusion, and only one had severe effusion. One patient showed signs of tamponade. Six patients from each group died, with a total of 12 fatalities, of which five had pericardial effusion, and only one had severe effusion. Although emergent hemodialysis patients had higher BUN levels, it didn't correlate with pericardial effusion presence or severity. | Two hundred fifty-one ESRD patients. | 2015 | Chang & Aisenberg. (31) |
| 4% were linked to uremia, which consist of cases of advanced renal diseases, whether in dialysis or pre-dialysis stages, after ruling out other potential causes. While initially presenting poorer outcomes, uremic cardiac tamponade showed an overall favorable prognosis. In contrast, patients with malignancies experienced more challenging outcomes. | One hundred thirty-six patients with the diagnosis of cardiac tamponade. | 2016 | Sánchez-Enrique et al.  (32) |
| The primary cause of effusive pericarditis in Africa was tuberculosis, accounting for 55% of cases. Uremia was considered as the etiology in 5% of patients. While HIV serology was positive in 18 (47%) of patients, only 5% of cases were attributed to HIV-related effusive pericarditis. | Thirty-eight patients with effusive pericarditis. | 2016 | Pio et al. (33) |
| Case Group: This consisted of 84 CKD patients at Stages 4 and 5 with pericardial effusion, of whom 44% were undergoing dialysis. Among them, 46% displayed moderate to large pericardial effusion. Control Group: There were 61 patients with the same CKD stage. Among them, 34% were under dialysis and did not have pericardial effusion. In the multivariate analyses findings revealed that a higher heart rate (OR: 1.290 per 10 bpm), elevated potassium level (OR: 1.949 per 1‐mEq/L), and lower corrected calcium (OR: 1.33 per 1‐mg/dL) were identified as independent factors predicting the presence of any amount of pericardial effusion. They also reported that a heart rate above 100 bpm, potassium level above 5 mEq/L, and corrected calcium below 8 mg/dL are highly specific in diagnosing pericardial effusion. Additionally, a corrected calcium level below 8 mg/dL demonstrated 95% specificity for moderate and large pericardial effusion when compared to the group with no effusion. Furthermore, in a subgroup analysis of patients who hadn't undergone dialysis yet, corrected calcium emerged as the sole independent factor predicting pericardial effusion. Notably, their study did not find an association between pericardial effusion and a worse short-term prognosis. | CKD patients at Stages 4 and 5 with pericardial effusion. | 2018 | Ravi et al.  (34) |
| The cause of pericardial effusion was attributed to uremia in 39% (220 out of 553) of cases. | Five hundred fifty-three patients underwent pericardial drainage. | 2019 | Colak et al. (35) |
| In an outpatient setting, a total of 2,820 patients with End-Stage Renal Disease (ESRD) underwent evaluation. Among these, 79.5% were receiving dialysis at the time of echocardiography. Exclusions were made for patients with concurrent medical conditions that could account for pericardial effusion. Out of the 2,820 patients, 54 exhibited moderate to large pericardial effusion.  A comparative analysis was conducted with ESRD patients lacking pericardial effusion. Among the 54 patients with pericardial effusion: 38 were undergoing intermittent dialysis.  41 had moderate pericardial effusion.  13 had severe pericardial effusion. 7 experienced tamponade.  The duration of hemodialysis (1.1 vs. 2.8 years) appeared to confer protection against the development of moderate and large pericardial effusion.   Crucially, a diagnosis of pericardial effusion did not correlate with a poorer prognosis. The 10-year survival rate was reported to be 87%. | Two thousand eight hundred twenty patients with ESRD. | 2020 | Ashraf et al. (36) |

1. Guild WR, Bray G, Merrill JP. Hemopericardium with cardiac tamponade in chronic uremia. The New England journal of medicine. 1957;257(5):230-1.

2. Keith NM, Burchell HB, Baggenstoss AH. Electrocardiographic changes in uremia associated with a high concentration of serum potassium: Report of three cases. American Heart Journal. 1944;27(6):817-44.

3. Langendorf R, Pirani CL. The heart in uremia: An electrocardiographic and pathologic study. American Heart Journal. 1947;33(3):282-307.

4. Symons HS, Wrong OM. URAEMIC PERICARDITIS WITH CARDIAC TAMPONADE: A REPORT OF FOUR CASES. British medical journal. 1964;1(5383):605-6.

5. Goodner CJ, Brown H. REPORT OF TWO CASES OF CARDIAC TAMPONADE IN UREMIC PERICARDITIS. Journal of the American Medical Association. 1956;162(16):1459-61.

6. Baldwin JJ, Edwards JE. Uremic pericarditis as a cause of cardiac tamponade. Circulation. 1976;53(5):896-901.

7. Merikas G, Samartzis M, Marketos S. Massive Cardiac Tamponade in Uremic Pericarditis with Complete Recovery. New England Journal of Medicine. 1962;266(21):1089-91.

8. Beaudry C, Nakamoto S, Kolff WJ. Uremic pericarditis and cardiac tamponade in chronic renal failure. Annals of internal medicine. 1966;64(5):990-5.

9. Alfrey AC, Goss JE, Ogden DA, Vogel JH, Holmes JH. Uremic hemopericardium. The American journal of medicine. 1968;45(3):391-400.

10. Krayenbühl HP, Turina J, Forouzan A. [Diagnosis and differential diagnosis of pericardial effusion]. Schweizerische medizinische Wochenschrift. 1976;106(12):393-400.

11. Santos GH, Frater RW. The subxiphoid approach in the treatment of pericardial effusion. The Annals of thoracic surgery. 1977;23(5):467-70.

12. D'Cruz IA, Bhatt GR, Cohen HC, Glick G. Echocardiographic detection of cardiac involvement in patients with chronic renal failure. Archives of internal medicine. 1978;138(5):720-4.

13. Matsumoto J, MURATA T, SATO S, ICHIHARA T, HARUYAMA T, URUGA K, et al. Echocardiographic Diagnosis, Classification, and Dialysis Treatment of Uremic Pericardial Effusion. The Tohoku Journal of Experimental Medicine. 1980;130(3):265-72.

14. Yoshida K, Shiina A, Asano Y, Hosoda S. Uremic pericardial effusion: detection and evaluation of uremic pericardial effusion by echocardiography. Clin Nephrol. 1980;13(6):260-8.

15. Prager RL, Wilson CH, Bender HW, Jr. The subxiphoid approach to pericardial disease. The Annals of thoracic surgery. 1982;34(1):6-9.

16. Frommer JP, Young JB, Ayus JC. Asymptomatic pericardial effusion in uremic patients: effect of long-term dialysis. Nephron. 1985;39(4):296-301.

17. Little AG, Kremser PC, Wade JL, Levett JM, DeMeester TR, Skinner DB. Operation for diagnosis and treatment of pericardial effusions. Surgery. 1984;96(4):738-44.

18. Leehey DJ, Daugirdas JT, Popli S, Gandhi VC, Pifarré R, Ing TS. Predicting need for surgical drainage of pericardial effusion in patients with end-stage renal disease. The International journal of artificial organs. 1989;12(10):618-25.

19. Palatianos GM, Thurer RJ, Pompeo MQ, Kaiser GA. Clinical Experience With Subxiphoid Drainage of Pericardial Effusions. The Annals of thoracic surgery. 1989;48(3):381-5.

20. Achari V, Thakur AK. Echocardiographic detection of cardiac involvement in chronic renal failure. J Assoc Physicians India. 1989;37(7):434-6.

21. Wall TC, Campbell PT, O'Connor CM, Van Trigt P, Kenney RT, Sheikh KH, et al. Diagnosis and management (by subxiphoid pericardiotomy) of large pericardial effusions causing cardiac tamponade. The American journal of cardiology. 1992;69(12):1075-8.

22. Robles R, Piñero A, Luján JA, Fernández JA, Torralba JA, Acosta F, et al. Thoracoscopic partial pericardiectomy in the diagnosis and management of pericardial effusion. Surgical endoscopy. 1997;11(3):253-6.

23. Vayre F, Lardoux H, Chikli F, Pezzano M, Bourdarias JP, Koukoui F, et al. [Evaluation of echo-guided pericardiocentesis in cardiac tamponade]. Archives des maladies du coeur et des vaisseaux. 1998;91(1):13-20.

24. Dogan R, Demircin M, Sarigül A, Ciliv G, Bozer AY. Diagnostic value of adenosine deaminase activity in pericardial fluids. The Journal of cardiovascular surgery. 1999;40(4):501-4.

25. Bastian A, Meißner A, Lins M, Siegel EG, Möller F, Simon R. Pericardiocentesis: differential aspects of a common procedure. Intensive Care Medicine. 2000;26(5):572-6.

26. Becit N, Ozyazicioğlu A, Ceviz M, Karakelleoğlu S, Karapolat S, Koçak H. Clinical experience with subxiphoid pericardiostomy in the management of pericardial effusions: a study of 240 cases. The Journal of international medical research. 2003;31(4):312-7.

27. Ben-Horin S, Bank I, Guetta V, Livneh A. Large symptomatic pericardial effusion as the presentation of unrecognized cancer: a study in 173 consecutive patients undergoing pericardiocentesis. Medicine (Baltimore). 2006;85(1):49-53.

28. Gümrükçüoğlu HA, Akyol A, Tuncer M, Güneş Y, Beğenik H, Akdağ S, et al. [Clinical and laboratory features of patients with pericardial effusion]. Turk Kardiyol Dern Ars. 2010;38(7):473-9.

29. Zahiti B, Bakalli A, Gorani D, Hasanxhekaj V. The importance of echocardiographic evaluation of pericardial effusion associated with mitral valve pseudoprolapse in patients with chronic renal failure. Saudi journal of kidney diseases and transplantation : an official publication of the Saudi Center for Organ Transplantation, Saudi Arabia. 2011;22(5):994-7.

30. Bataille S, Brunet P, Decourt A, Bonnet G, Loundou A, Berland Y, et al. Pericarditis in uremic patients: serum albumin and size of pericardial effusion predict drainage necessity. J Nephrol. 2015;28(1):97-104.

31. Chang KW, Aisenberg GM. Pericardial Effusion in Patients with End-Stage Renal Disease. Texas Heart Institute journal. 2015;42(6):596.

32. Sánchez-Enrique C, Nuñez-Gil IJ, Viana-Tejedor A, De Agustín A, Vivas D, Palacios-Rubio J, et al. Cause and Long-Term Outcome of Cardiac Tamponade. The American journal of cardiology. 2016;117(4):664-9.

33. Pio M, Afassinou YM, Pessinaba S, Mossi KE, Kotosso A, Baragou S, et al. [Effusive pericarditis: clinical and etiological aspects in Lomé]. Medecine et sante tropicales. 2016;26(1):92-6.

34. Ravi V, Iskander F, Saini A, Brecklin C, Doukky R. Clinical predictors and outcomes of patients with pericardial effusion in chronic kidney disease. Clin Cardiol. 2018;41(5):660-5.

35. Colak A, Becit N, Kaya U, Ceviz M, Kocak H. Treatment of Pericardial Effusion Through Subxiphoid Tube Pericardiostomy and Computerized Tomography- or Echocardiography - Guided Percutaneous Catheter Drainage Methods. Brazilian journal of cardiovascular surgery. 2019;34(2):194-202.

36. Ashraf H, Lee H, Tran KH, Agasthi P, Keddis MT, Unzek S, et al. Prevalence and Outcomes of Pericardial Effusion in Kidney Transplant Candidates. The American journal of cardiology. 2020;132:140-6.
